# Trajectories of Physical Function and Behavioral, Psychological, and Social Well-Being in a Cohort of Swedish Older Adults

**DOI:** 10.1093/geroni/igad040

**Published:** 2023-05-04

**Authors:** Marguerita Saadeh, Xin Xia, Eline Verspoor, Anna-Karin Welmer, Serhiy Dekhtyar, Davide L Vetrano, Laura Fratiglioni, René J F Melis, Amaia Calderón-Larrañaga

**Affiliations:** Aging Research Center, Department of Neurobiology, Care Sciences and Society, Karolinska Institutet & Stockholm University, Solna, Sweden; Aging Research Center, Department of Neurobiology, Care Sciences and Society, Karolinska Institutet & Stockholm University, Solna, Sweden; Department of Geriatric Medicine, Radboudumc Alzheimer Centrum, Radboud University Medical Center, Nijmegen, The Netherlands; Radboud Institute for Health Sciences, Radboud University Medical Center, Nijmegen, The Netherlands; Aging Research Center, Department of Neurobiology, Care Sciences and Society, Karolinska Institutet & Stockholm University, Solna, Sweden; Division of Physiotherapy, Department of Neurobiology, Care Sciences and Society, Karolinska Institutet, Stockholm, Sweden; Aging Research Center, Department of Neurobiology, Care Sciences and Society, Karolinska Institutet & Stockholm University, Solna, Sweden; Aging Research Center, Department of Neurobiology, Care Sciences and Society, Karolinska Institutet & Stockholm University, Solna, Sweden; Stockholm Gerontology Research Center, Sweden; Aging Research Center, Department of Neurobiology, Care Sciences and Society, Karolinska Institutet & Stockholm University, Solna, Sweden; Stockholm Gerontology Research Center, Sweden; Department of Geriatric Medicine, Radboudumc Alzheimer Centrum, Radboud University Medical Center, Nijmegen, The Netherlands; Radboud Institute for Health Sciences, Radboud University Medical Center, Nijmegen, The Netherlands; Aging Research Center, Department of Neurobiology, Care Sciences and Society, Karolinska Institutet & Stockholm University, Solna, Sweden; Stockholm Gerontology Research Center, Sweden

**Keywords:** Longitudinal studies, Older adults, Successful aging

## Abstract

**Background and Objectives:**

Successful aging has been described as a multifactorial and dynamic process. The aims of the study were to detect aging trajectories of physical function and behavioral, psychological, and social well-being; and to explore the correlations between functional versus well-being trajectories by age group.

**Research Design and Methods:**

Data were gathered from the Swedish National Study on Aging and Care in Kungsholmen (*N* = 1,375). Subjects’ physical function was assessed through walking speed and chair-stand tests, behavioral well-being through participation in mental and physical activities, psychological well-being through life satisfaction and positive affect, and social well-being through social connections and support. All exposures were standardized (*z*-scores). Linear mixed models were used to estimate trajectories of physical function and well-being over a 12-year follow-up.

**Results:**

The steepest declines were seen for physical function (relative change [RC] in *z*-scores across ages; RC = 3.01), followed by behavioral well-being (RC = 2.15), psychological well-being (RC = 2.01), and social well-being (RC = 0.76). Correlations between physical function and the different well-being domains were weak, especially for slopes. Stronger intercept correlations were observed among the oldest—compared to the youngest-old, especially with behavioral (*r* = 0.39 vs *r* = 0.24) and psychological (*r* = 0.33 vs *r* = 0.22) well-being.

**Discussion and Implications:**

Physical function declines the fastest throughout aging. The different well-being domains decline at a slower rate, which may be a possible sign of compensation against age-related functional decline, especially among the youngest-old, for whom discordances between physical function and the different well-being domains were more common.

**Translational Significance:** The current study provides important insights into the multifactorial and dynamic nature of aging by looking at longitudinal trajectories of physical functional and behavioral, psychological, and social well-being. Our findings highlight that, even if all indicators worsen with increasing age, physical function declines the fastest. Behavioral, psychological, and social well-being decline at a slower rate and may act as potential compensatory mechanisms of physical health deterioration, especially among the youngest-old. These findings should encourage researchers to better understand such compensatory mechanisms, and health care professionals and public health specialists to promote health across different dimensions of older people’s lives.

Maintaining good physical functioning is a fundamental pillar of successful aging, as it is considered the most valued goal among older adults (Beswick et al., 2008) and an acknowledged proxy of biological aging given its association with major health endpoints including disability, hospitalization, and death (Cawthon et al., 2012; Ferrucci et al., 2018; Pavasini et al., 2016; Vermeulen et al., 2011). Nevertheless, the paradigm viewing good physical health as the main indicator of successful aging has come under heavy scrutiny in recent years (Rowe & Kahn, 2015). Several definitions of successful aging imply that people may reach advanced age without any age-related diseases and/or functional deterioration (Michel et al., 2021; Stowe & Cooney, 2015). Such physiologically oriented definitions fail to capture the multifaceted nature and vast heterogeneity of aging phenotypes, and predetermine most older adults to fail at aging successfully (Hank, 2011). Indeed, several centenarian studies have shown that it is inherently difficult, if not impossible, to reach advanced age without chronic diseases and any physical impairment (Herr et al., 2018; Vetrano et al., 2020).

Based on older adults’ own perspectives, behavioral well-being (i.e., participation in physically and mentally stimulating leisure activities), psychological well-being (i.e., presence of positive emotions and life satisfaction), and social well-being (i.e., ability to communicate with others and build meaningful relationships) have been endorsed as being equally important domains to be preserved for successful aging (Phelan et al., 2004). This multidimensional model allows to better capture the heterogeneity among aging individuals and reflects the possibility of aspiring to and achieving well-being in old age despite age-related diseases and disabilities. Of note, it has been suggested that behavioral, psychological, and social aspects could potentially enable individuals, and especially older adults, to cope with the aging-inherent physical decline and, hence, age successfully (Young et al., 2009).

Older persons seem to compensate and adapt to functional deficits accumulated across the life span through goal-directed behaviors and/or engagement in valued activities (Jonker et al., 2009; Remillard et al., 2019; Young et al., 2009). This is in line with the selection, optimization, and compensation model, which describes how individuals adapt to age-related declines in terms of, among others, physical function (Baltes, 1997). Indeed, older adults’ well-being cannot simply be classified as consistently good or bad, because discordances across different domains might reflect a combination of gains and losses affecting individuals differently. For instance, in a recent study, we have shown that older individuals with either high social or psychological well-being seem to preserve their physical function for longer, as compared to those with both low psychological and social well-being (Saadeh et al., 2020). Another study showed that older people engaging in goal-oriented compensatory activities report high levels of well-being in spite of physical disability (Carpentieri et al., 2017).

Few studies have examined longitudinal trajectories of physical function versus behavioral, psychological, and social well-being, despite the evidence showing that both baseline values, as well as changes in all of these domains, are associated with major health endpoints (Hirsch et al., 2012). This hinders a proper understanding of the concordance, discordance, and compensatory potential between physical function versus the different well-being domains. Furthermore, it has been hypothesized that subjects’ compensatory potential decreases with age due, in part, to the increasing biological and physical constraints (Freund & Baltes, 2002), which calls for more nuanced age-stratified analyses.

The specific aims of this study were, therefore, (a) to describe the aging trajectories of physical function and behavioral, psychological, and social well-being; and (b) to examine the correlation between physical function versus the different well-being domains by age group, in terms of baseline values and linear change.

## Material and Methods

### Study Population

Data were gathered from the Swedish National Study on Aging and Care in Kungsholmen (SNAC-K; https://www.snac-k.se/). SNAC-K is an ongoing population-based study including community-dwelling and institutionalized adults aged 60 years or older, residing in the Kungsholmen district of Stockholm, Sweden. A random sample of individuals from 11 age cohorts (ages 60, 66, 72, 78, 81, 84, 87, 90, 93, 96, and ≥99 years) were invited to participate in the study between 2001 and 2004 (baseline). The baseline SNAC-K population included 3,363 individuals (73.3% participation rate) who have been followed up regularly: every 6 years for the young-old cohorts (<78 years) and every 3 years for the older cohorts (≥78 years). At each study wave, physicians, nurses, and psychologists conduct extensive clinical examinations, interviews, and assessments following standard procedures. SNAC-K data are linked to the National Patient Register and the Swedish Cause of Death Register to obtain information on medical history and vital status.

This study included data from SNAC-K baseline and subsequent four follow-ups covering a maximum of 12 years. Out of the 3,363 participants from SNAC-K baseline, we excluded 1,393 (41.4%) participants with missing data on any well-being domain at baseline. We further excluded 595 participants with no follow-up data on each and every well-being domain (30.2% of the remaining sample). After applying the exclusion criteria, we ended up with 1,375 participants with information on all well-being domains at baseline and at least one follow-up. SNAC-K was approved by the Swedish Ethical Review Authority in Stockholm, and written informed consent was obtained from participants or their next of kin.

### Study Variables

#### Assessment of physical function

Participants’ mobility was measured by means of the walking speed assessed by trained nurses, whereby participants were asked to walk 6 or 2.4 m, at a self-selected speed and using a walking aid if needed. The length of the walk was determined by asking participants how fast they normally walked (i.e., normal/fast walkers did the longer walk and slow/very slow walkers did the shorter walk). It was reported as meters per second (m/s). Participants’ muscle strength was measured through the chair-stand test, which was performed by asking participants to fold their arms across their chest and stand up from a seated position five times consecutively as quickly as possible. It was reported in seconds. Chair-stand scores were reversed to enable their interpretation in the same direction as walking speed. The scores for both tests were standardized (i.e., *z*-scores) based on the baseline means and standard deviations (*SD*s), and a physical function score was computed by averaging both standardized scores.

#### Assessment of behavioral well-being

Participants were asked to specify the type and frequency (i.e., monthly, weekly, less frequently, never) of the leisure activities they engaged in. We grouped the reported activities into mental and physical according to the category they predominantly belonged to, following a procedure described elsewhere (Köhncke et al., 2016). Mental leisure activities included using the internet/playing computer games, painting/drawing/working with clay/pottery, carrying out car or mechanical repairs, and reading books. Physical leisure activities included gardening, hiking in the forest/pick berries or mushrooms, hunting/fishing, doing home repairs, and carrying out light exercise (e.g., walking along roads/in parks, walking in the woods, short bicycle rides, light aerobics, golf) or moderate to intense exercise (e.g., jogging, long power walks, heavy-duty gardening, long bicycle rides, high-intensity aerobics, long-distance ice skating, swimming, ball sports other than golf, or other similar activities). Once again, the scores for each type of activity were standardized (i.e., *z*-scores) based on the baseline means and *SD*s, and a behavioral well-being score was computed by averaging the standardized scores of mental and physical leisure activity participation.

#### Assessment of psychological well-being

Life satisfaction was assessed through the self-reported Life Satisfaction Index A (LSI-A) that captures five different components: zest versus apathy, resolution and fortitude, congruence between desired and achieved goals, positive self-concept, and mood tone. The LSI-A consists of 20 items with an “agree,” “disagree,” or “uncertain” response (range 0–100). A high score indicates that the person takes pleasure from the round of activities that constitutes his or her everyday life, regards life as meaningful and resolutely accepts life as it has been, feels he or she has succeeded in achieving his or her major goals, holds a positive self-image, and maintains happy and optimistic attitudes and mood (Saadeh et al., 2020; Straatmann et al., 2020). Positive affect was measured based on the following affective features: active, inspired, determined, alert, and enthusiastic. Respondents were asked to report whether and to what extent they had felt in the above-mentioned affective states during the last 4 weeks. The response options were “not at all,” “a little,” “somewhat,” “quite a bit,” and “very much” (range 5–25; Saadeh et al., 2020; Straatmann et al., 2020). As done earlier, the scores for life satisfaction and positive affect were standardized (i.e., *z*-scores) based on the baseline means and *SD*s, and a psychological well-being score was computed by averaging both standardized scores.

#### Assessment of social well-being

Participants’ social connections were assessed by asking them about their marital status, cohabitation status, parenthood, friendships, and the frequency of direct or remote contact with parents, children, relatives, neighbors, and friends (Hanson et al., 1997; Saadeh et al., 2020). Participants’ social support was measured by asking them about their satisfaction concerning their contacts with subjects from their social network, perceived material and psychological support, sense of affinity with potential associations, relatives, and residence area, and feeling part of a group of friends (Hanson et al., 1997; Saadeh et al., 2020). As done for the rest of the well-being domains, each of these measures was standardized (i.e., *z*-scores) based on the baseline means and *SD*s, and a social well-being score was computed by averaging the scores for both social connections and social support.

### Statistical Analysis

Linear mixed models were used to estimate the baseline values and longitudinal changes in physical function and behavioral, psychological, and social well-being over the 12-year follow-up. We tested two models for each of these functional and well-being domains that included baseline age, follow-up time, age*follow-up time (Model 1), and age, sex, follow-up time, age*follow-up time, sex*follow-up time (Model 2) as fixed effects. *Follow-up time* was included as a random effect. The best-fit model was chosen based on the following goodness-of-fit indices: Akaike Information Criterion, Bayesian Information Criterion, and likelihood ratio test. Functional and well-being trajectories were displayed according to participants’ birth cohort, and the relative changes (RCs) within each well-being domain were measured by calculating the differences in average *z*-scores between the baseline assessment of the youngest cohort and the last follow-up assessment of the oldest cohort.

Correlations among intercepts and slopes for physical function versus the different well-being domains for the total study population and by baseline age categories (<78 and ≥78 years) were tested using Spearman’s correlations. The age cutoff of 78 was chosen based on the age categories linked to the design of SNAC-K, and its proximity to the definition of “oldest-old” by the American Geriatrics Society (i.e., individuals aged 80 years and above). Last, we categorized the individual-level random estimates of intercepts and slopes into low and high according to the medians. We further cross-classified individuals’ levels of physical function with their levels of behavioral, psychological, and social well-being, respectively, also by baseline age categories.

All analyses were performed using R version 3.6.1 and Stata version 15.1 with the level of statistical significance set at *p* < .05.

## Results

The study population consisted of 1,375 individuals with a mean age of 70 years (*SD*: 8.9) at baseline (Table 1). The majority were female (62.1%), with at least high school level education (90.4%). The study sample had a median of three chronic diseases (IQR: 2; 5) and a median MMSE (Mini-Mental State Examination) score of 29 (IQR: 29; 30) at baseline. Participants aged 78 years and older performed worse on physical function tests (i.e., walking speed and chair stands) and reported lower behavioral, psychological, and social well-being as compared to those younger than 78 years. Around a third of the study population (27.6%) died during the follow-up (Table 1). On average, study participants were followed up for 10.1 years (*SD*: 2.6), which corresponds to an average of 3.3 (*SD*: 0.8) observations per participant. Compared to SNAC-K participants excluded from the study, included participants were younger, more likely to be female, have a higher educational level, and presented with a lower number of chronic diseases and higher MMSE score at baseline (Supplementary Table 1).

**Table 1.** Baseline Characteristics of the Study Population

Characteristic	Total population (*n* = 1,375)	<78 years (*n* = 971)	≥78 years (*n* = 404)	*p* Value
Age, mean (SD)	69.9 (8.9)	65.0 (4.7)	81.7 (4.3)	**<.001**
Sex, *n* (%)				
Male	521 (37.9)	388 (40.0)	133 (32.9)	**<.001**
Female	854 (62.1)	583 (60.0)	271 (67.1)	
Education, *n* (%)				
Elementary	132 (9.6)	65 (6.7)	67 (16.6)	**<.001**
High school	663 (48.2)	437 (45.0)	226 (55.9)	
University	580 (42.2)	469 (48.3)	111 (27.5)	
Number of chronic diseases, median (IQR)	3 (2; 5)	2 (1; 4)	4 (3; 6)	**<.001**
MMSE score, median (IQR)	29 (29; 30)	30 (29; 30)	29 (28; 30)	**<.001**
Physical function, median *z*-score (IQR)	0.57 (0.3; 0.9)	0.62 (0.5; 0.9)	0.26 (−0.3; 0.5)	**<.001**
Behavioral well-being, median *z*-score (IQR)	0.32 (-0.2; 0.9)	0.58 (0.1; 1.1)	−0.26 (−0.6; 0.2)	**<.001**
Psychological well-being, median *z*-score (IQR)	0.27 (−0.3; 0.8)	0.48 (−0.1; 0.8)	−0.08 (−0.7; 0.4)	**<.001**
Social well-being, median *z*-score (IQR)	0.24 (−0.1; 0.5)	0.29 (−0.1; 0.6)	0.14 (−0.2; 0.4)	**<.001**
Number of deaths by the end of follow-up, *n* (%)	379 (27.6)	116 (12.0)	263 (65.1)	**<.001**

*Notes*: IQR = inter quartile range; MMSE = Mini-Mental State Examination; *SD* = standard deviation. Chronic diseases were operationalized according to a methodology described previously (number range: 0–60; Calderón-Larrañaga et al., 2017), and cognitive function using the MMSE (score range: 0–30, higher scores indicate better global cognition).

**Figure 1.**
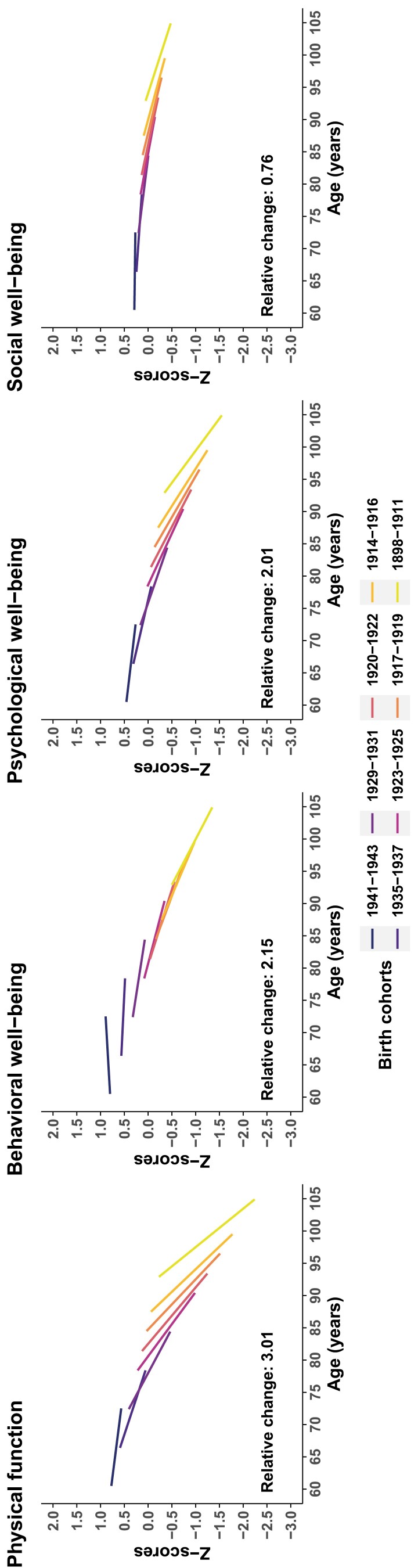
Aging trajectories of physical function and behavioral, psychological, and social well-being. Relative change *=* the difference between the average baseline *z*-score for the youngest cohort and the average 12-year *z*-score for the oldest cohort.

Model 2 was chosen for all functional and well-being domains based on goodness-of-fit parameters (Supplementary Tables 2 and 3). As shown in Figure 1, all domains declined over time, even if the magnitude of decline varied across domains. The RC (i.e., the difference between the average baseline *z*-score for the youngest cohort and the average 12-year *z*-score for the oldest cohort) was highest for physical function (RC = 3.01), followed by a moderate decline for behavioral (RC = 2.15) and psychological (RC = 2.01) well-being, and with the lowest decline observed for social well-being (RC = 0.76). Declines tended to become steeper at older ages, especially for physical function. Declines in intercepts or slopes were minimal for social well-being. Similar aging trajectories were observed in men and women across the individual components of physical function and the different well-being domains (Supplementary Figure 1).

The correlations between the intercepts and slopes of physical function versus behavioral, psychological, and social well-being are shown in Table 2. Correlations were stronger among oldest- versus youngest-old subjects between the intercepts of physical function and behavioral well-being (*r* = 0.24 among those <78 years vs *r* = 0.39 among those ≥78 years) as well as the intercepts of physical function and psychological well-being (*r* = 0.22 among those <78 years vs *r* = 0.33 among those ≥78 years). Weaker correlations were observed between the intercepts of physical function and social well-being for both age groups (*r* = 0.16). Concerning the slopes, correlations were, in general, very weak. The strongest correlations were observed between physical function and psychological well-being (*r* = 0.22 among those <78 years vs *r* = 0.17 among those ≥78 years).

**Table 2.** Correlations (*Spearman*) and Statistical Significance (*p Values*) Between the Intercepts and Slopes of Physical Function Versus Behavioral, Psychological, and Social Well-Being in the Total Population and by Age Group

Variable	Physical function		
	Total population (*n* = 1,375)	<78 years (*n* = 971)	≥78 years (*n* = 404)
Intercept			
Behavioral well-being	0.29 (<0.001)	0.24 (<0.001)	0.39 (<0.001)
Psychological well-being	0.26 (<0.001)	0.22 (<0.001)	0.33 (<0.001)
Social well-being	0.16 (<0.001)	0.16 (<0.001)	0.16 (0.001)
Slope			
Behavioral well-being	0.12 (<0.001)	0.14 (<0.001)	0.06 (0.194)
Psychological well-being	0.21 (<0.001)	0.22 (<0.001)	0.17 (0.001)
Social well-being	0.15 (<0.001)	0.18 (<0.001)	0.12 (0.019)

As shown in Table 3, among individuals younger than 78 years and with low physical function at baseline, almost half had high behavioral (44.8%), psychological (45.6%), and social (45.0%) well-being. However, such discordances were less frequent among subjects 78 years and older, especially for behavioral and psychological well-being (29.7% and 35.9%, respectively). When looking at the distribution of slopes among subjects younger than 78 years with high functional decline (see Table 4), 44.5%, 43.1%, and 41.6% showed low declines in behavioral, psychological, and social well-being, respectively. Such discordances were of similar magnitude among those 78 years and older.

**Table 3.** Distribution (Row Percentages) of Individuals’ Intercepts Across Physical Function and the Different Well-Being Domains by Age Group

Physical Function	Behavioral well-being		Psychological well-being		Social well-being	
	Low	High	Low	High	Low	High
<78 years						
Low	263 (55.3)	213 (44.8)	259 (54.4)	217 (45.6)	262 (55.0)	214 (45.0)
High	192 (38.8)	303 (61.2)	214 (43.2)	281 (56.8)	216 (43.6)	279 (56.4)
≥78 years						
Low	149 (70.3)	63 (29.7)	136 (64.2)	76 (35.9)	119 (56.1)	93 (43.9)
High	84 (43.8)	108 (56.3)	79 (41.2)	113 (58.9)	91 (47.40)	101 (52.6)

*Note*: Individual-level random estimates of intercepts were categorized into low and high according to the medians.

**Table 4.** Distribution (Row Percentages) of Individuals’ Slopes Across Physical Function and the Different Well-Being Domains by Age Group

Physical Function	Behavioral well-being		Psychological well-being		Social well-being	
	High decline	Low decline	High decline	Low decline	High decline	Low decline
<78 years						
High decline	251 (55.5)	201 (44.5)	257 (56.9)	195 (43.1)	264 (58.4)	188 (41.6)
Low decline	238 (45.9)	281 (54.1)	222 (42.8)	297 (57.2)	234 (45.1)	285 (54.9)
≥78 years						
High decline	115 (48.7)	121 (51.3)	133 (56.4)	103 (43.6)	117 (49.6)	119 (50.4)
Low decline	84 (50.0)	84 (50.0)	76 (45.2)	92 (54.8)	73 (43.5)	95 (56.6)

*Note*: Individual-level random estimates of slopes were categorized into low and high according to the medians.

## Discussion

In this large, population-based cohort study of older adults aged 60 years and above, we found that physical function and the different well-being domains declined at different rates, with the steepest declines seen for physical function followed by behavioral, psychological, and social well-being. Correlations between physical function and the different well-being domains were weak, particularly for slopes. Stronger intercept correlations were observed among the oldest-old compared to the youngest-old, especially with behavioral and psychological well-being. Accordingly, discordances between baseline physical function and the different domains of well-being were more common among the youngest- than the oldest-old.

There is a clear lack of literature looking comparatively at how physical function and different domains of well-being decline over time among older adults, which makes it hard to compare our results with other studies. A recent systematic review supports our finding that physical function declines at a faster rate, potentially preceding declines in behavioral, psychological, and social domains (Bortone et al., 2021; Young et al., 2009). The decline in physical function starts around the age of 60, with the steepest declines observed after the age of 80 years due to age-related processes affecting physical independence (Bohannon & Williams Andrews, 2011). In accordance with our results, a study of 1,398 participants in the Swedish Adoption/Twin Study (SATSA) concluded that there is a decline in older people’s participation in physical and mental leisure activities after the age of 65, which seems to accelerate in advanced old age (Finkel et al., 2018). Other longitudinal studies of American older adults also reported that leisure activities decline relatively early in the life course and at increasingly steep rates among older age groups (Janke et al., 2006; Shaw et al., 2010). It has been argued that this could result from the preceding decline in physical function (Hassett et al., 2021) or, on a positive note, it could also be due to the tendency of older adults to select fewer but more meaningful leisure activities to adapt to changes occurring as they age (Kleiber et al., 2008). Similarly, psychological well-being has been shown to be relatively stable among youngest-old adults but to decline rapidly among the oldest-old, which could be explained by the increased dependency, health problems, and death-related thoughts driving late-life changes in psychological well-being (Gerstorf et al., 2010; Lim et al., 2017). Social well-being has been shown to remain quite stable over time as compared to other domains (Finkel et al., 2018), which has been previously explained by the continuity theory that argues that individuals become used to a certain social network throughout their lives and attempt to maintain it even when transitioning to old age (Cornwell et al., 2008).

It is well known that behavioral, psychological, and social resources, such as leisure activity participation, high life satisfaction, and rich social network, have beneficial effects on the physical health of older adults (Dedeyne et al., 2017; Feng et al., 2017; Kim et al., 2021). A study of 5,451 older adults with a mean age of 67 years showed that engagement in leisure time activity in later life is associated with a slower speed of physical function decline (Chen et al., 2016). Another study of 3,577 older adults found that those with high levels of psychological well-being (i.e., purpose-in-life, resilience, optimism, internal locus of control) and social connections had better physical functioning, health status, and lower health care utilization (Musich et al., 2021). It has also been argued that when an older person’s function is declining, friends and family may offer sufficient support to keep the person safe and in reasonably good health (Zammit et al., 2014). Adopting healthy behaviors and psychosocial resources may help people cope with stressful life events and may play a modifying role in transitioning between stages in the disablement process and, thus, help preserve older adults’ independence (Cooper et al., 2011; Paggi et al., 2016; Sala et al., 2019). However, as people age, the accumulation of health deficits and their consequences may be increasingly difficult to reverse and/or compensate for (Mitnitski & Rockwood, 2015). In other words, in late old age, persons tend to experience losses in multiple domains, limiting the possibility for compensation. This is reflected in our results by the stronger baseline correlations observed between physical function and the different well-being domains in older- versus younger-old subjects. This may imply that persons can utilize behavioral, psychological, and social resources more efficiently in the earlier rather than in the later stages of old age, when individuals are more likely to already be affected by disability, to compensate for functional losses (Ailshire & Crimmins, 2011). Based on these findings, successful aging seems to be a process where different domains are bidirectionally associated and may thus play different roles at different phases of the aging process. In young-old age, behavioral, psychological, and social well-being may emerge as important protective factors against functional decline, but once older peoples’ physical function is impaired, behavioral, psychological, and social factors may also be affected and, therefore, protecting physical function from further decline may become a therapeutic target by itself, with the final aim of preserving behavioral, psychological, and social well-being.

The discordances between physical function and the different well-being domains observed in our study (especially in the youngest-old) further suggest that behavioral, psychological, and social factors may contribute to mitigate the negative impact of functional decline, but this remains to be tested. A study from seven multiple sclerosis centers in Italy assessing 168 participants concluded that well-being can coexist with ill-being, and that it can counterbalance the negative effects of diseases (Bassi et al., 2014). Similarly, a qualitative study including 49 middle-aged and older individuals with physical disability (i.e., spinal cord injury, multiple sclerosis, muscular dystrophy, or postpolio syndrome) concluded that successful aging can be achieved despite physical disability, and highlighted the importance of psychological resiliency and adaptation, autonomy, participation in valued activities, and social connectedness as tools to compensate for functional impairments (Molton & Yorkston, 2016). Even if some older people are considered “lucky agers” given that they suffer from minimal physical decline, most older people must adapt to physical loss in order to maintain well-being (Kahana et al., 2014). Although research is currently shifting toward acknowledging successful aging as a multidimensional construct, most studies have focused on the concordances instead of discordances between health and well-being. In this study, we show that discordances between physical function and different well-being domains are common among older people, and a possible sign of compensation, even among the oldest-old.

The strengths of our study include its longitudinal design with a long follow-up and a relatively large sample size. Moreover, we used objective indicators of physical function and subjective indicators of well-being assessed across multiple time points, providing us with reliable information on their temporal evolution. However, some limitations need to be acknowledged. Functional and well-being trajectories were assessed simultaneously, which prevents us from establishing any chronological order (and hence causality) concerning changes in any of the dimensions. SNAC-K is based on a relatively healthy and wealthy sample of participants and our sample moreover excluded subjects with missing data who were sicker, all of which limits the transferability of our results to other populations of older adults. It has been acknowledged that people with a low socioeconomic status develop functional limitations earlier in life, and have more difficulty to build the required resources to deal with such declines (Kok et al., 2016). Trajectories of cognitive function were not examined in our paper but deserve equal attention as the rest of domains. Nevertheless, physical and cognitive functions are closely and bidirectionally related (Clouston et al., 2013; Zammit et al., 2019). Information on behavioral, psychological, and social well-being indicators was self-reported, which might have led to misclassification bias among those with low MMSE scores (<24) or diagnosed with dementia. Still, only four participants (0.3% of the study sample) presented with such conditions at baseline. Last, the concepts of well-being and successful aging are constantly evolving, and it may thus be possible that our operationalizations do not always align with individuals’ own views and experiences.

The current study provides important insights into the multifactorial and dynamic nature of aging. In summary, even if all functional and well-being domains worsen with increasing age, physical function declines the fastest. Behavioral, psychological, and social well-being domains decline at a slower rate and may act as potential compensatory mechanisms of physical health deterioration, especially among the youngest-old. These findings should encourage researchers to better understand such compensatory mechanisms, and health care professionals and public health specialists to promote health across different dimensions of older people’s lives.

## Supplementary Material

igad040_suppl_Supplementary_Materials

## Data Availability

Data, analytic methods, and materials are available to other researchers for replication purposes. The study reported in the manuscript was not pre-registered. Data are from the SNAC-K project, a population-based study on aging and dementia (http://www.snac-k.se/). Access to these original data is available to the research community upon approval by the SNAC-K data management and maintenance committee. Applications for accessing these data can be submitted to Maria Wahlberg (Maria.Wahlberg@ki.se) at the Aging Research Center, Karolinska Institutet.
