# Supplementary material for: Trajectories of Physical Function and Behavioral, Psychological, and Social Well-Being in a Cohort of Swedish Older Adults
Source: Innov Aging. 2023 May 4;7(5):igad040. doi: 10.1093/geroni/igad040 (PMC10287187; doi:10.1093/geroni/igad040)
Supplement: igad040_suppl_Supplementary_Materials [file igad040_suppl_supplementary_materials.docx]

**Online Supplementary Material**

**Supplementary Figure 1.** Aging trajectories of individual indicators of physical function and behavioral, psychological, and social well-being by birth cohort and sex. The background grey lines represent individual predicted trajectories.

**
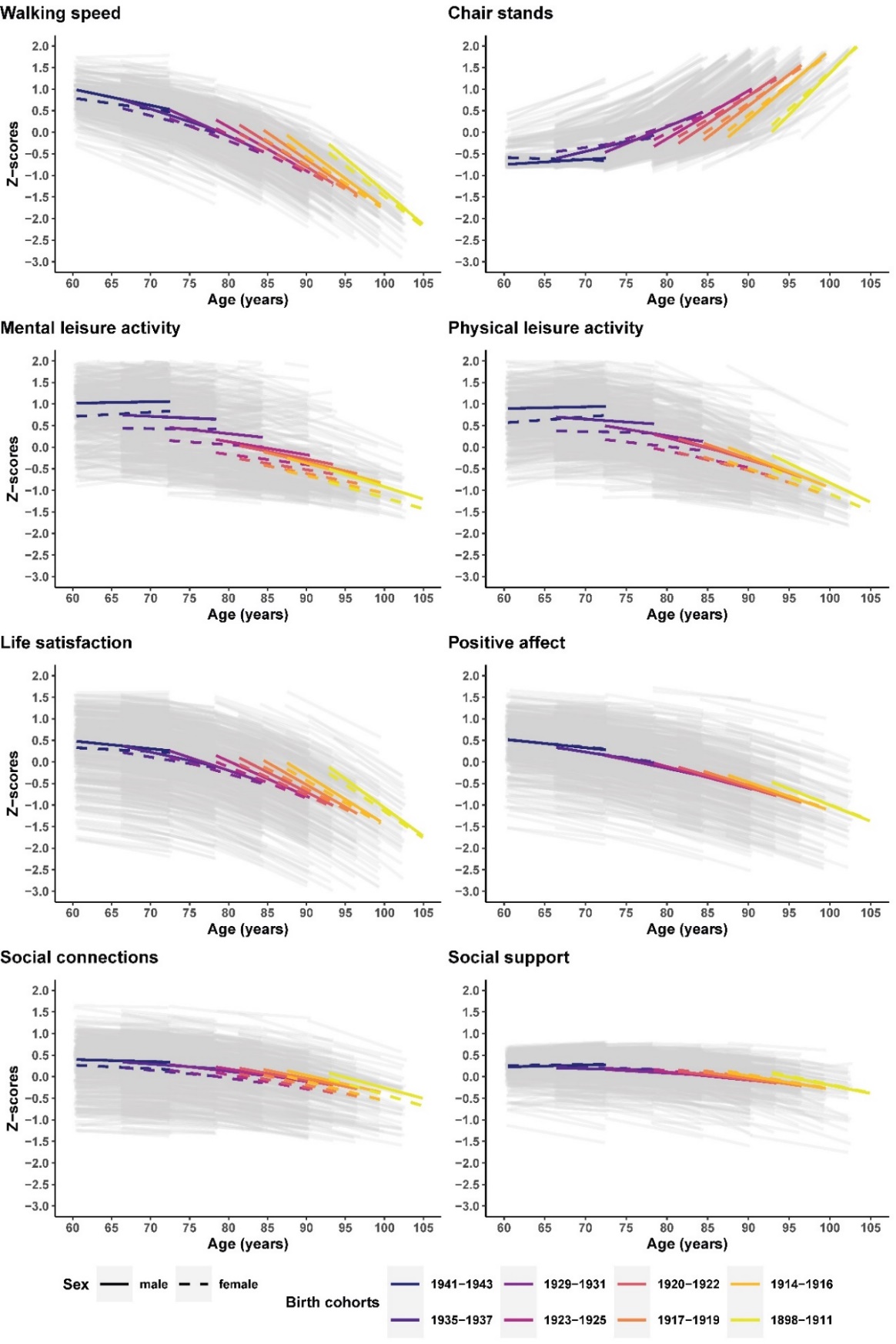
**

**Supplementary Table 1.** Comparisons between included and excluded individuals.

|  | **Excluded (n=1988)** | **Included (n=1375)** | **Total (N=3363)** |
| --- | --- | --- | --- |
| **Age** |  |  |  |
| Mean (SD) | 78.0 (11.4) | 69.9 (8.89) | 74.7 (11.2) |
| Min - Max | 60.0-104 | 60.0-96.5 | 60.0-104 |
| **Sex** |  |  |  |
| Female, n (%) | 1328 (66.8%) | 854 (62.1%) | 2182 (64.9%) |
| **Education** |  |  |  |
| Elementary, n (%) | 458 (23.0%) | 132 (9.6%) | 590 (17.5%) |
| High school, n (%) | 988 (49.7%) | 663 (48.2%) | 1651 (49.1%) |
| University, n (%) | 510 (25.7%) | 580 (42.2%) | 1090 (32.4%) |
| Missing, n (%) | 32 (1.6%) | 0 (0%) | 32 (1.0%) |
| **Number of chronic diseases** |  |  |  |
| Mean (SD) | 4.58 (2.60) | 3.30 (2.06) | 4.06 (2.48) |
| Min - Max | 0-16.0 | 0-12.0 | 0-16.0 |
| **MMSE score** |  |  |  |
| Mean (SD) | 25.9 (6.72) | 29.2 (0.969) | 27.2 (5.44) |
| Min - Max | 0-30.0 | 23.0-30.0 | 0-30.0 |
| Missing, n (%) | 27 (1.4%) | 0 (0%) | 27 (0.8%) |

*Notes*: SD = standard deviation.

**Supplementary Table 2.** Goodness-of-fit indices of linear mixed models for physical function and different well-being domains.

|  | **Physical function** | | |
| --- | --- | --- | --- |
|  | **AIC** | **BIC** | **LRT** |
| **Model 1** | 7847.847 | 7899.202 |  |
| **Model 2** | 7787.201 | 7870.652 | **<0.001** |
|  | **Behavioral well-being** | | |
|  | **AIC** | **BIC** | **LRT** |
| **Model 1** | 6815.34 | 6866.243 |  |
| **Model 2** | 6722.448 | 6805.166 | **<0.001** |
|  | **Psychological well-being** | | |
|  | **AIC** | **BIC** | **LRT** |
| **Model 1** | 8504.128 | 8554.798 |  |
| **Model 2** | 8496.159 | 8578.498 | **<0.001** |
|  | **Social well-being** | | |
|  | **AIC** | **BIC** | **LRT** |
| **Model 1** | 3246.213 | 3297.116 |  |
| **Model 2** | 3196.241 | 3278.958 | **0.011** |

*Notes:* AIC = Akaike Information Criterion; BIC = Bayesian Information Criterion; LRT = Likelihood-Ratio test. Fixed effects included *age, follow-up time, age*follow-up time* (Model 1), and *age, sex, follow-up time, age*follow-up time,* *sex*follow-up time* (Model 2). *Follow-up time* was included as a random effect in the models.

**Supplementary Table 3.** Fixed and random effect estimates of linear mixed models for physical function and different well-being domains.

| **Physical function** | | |
| --- | --- | --- |
| **Fixed effects** | **ꞵ coefficients (SE)** | **P-value** |
| Intercept | 2.732 (0.1154) | **<0.0001** |
| Age | -0.031 (0.0016) | **<0.0001** |
| Time | 0.256 (0.0145) | **<0.0001** |
| Sex | -0.176 (0.0303) | **<0.0001** |
| Age*time | -0.005 (0.0002) | **<0.0001** |
| Sex*time | 0.015 (0.0036) | **<0.0001** |
| **Random effects** |  | |
| Intercept | 0.362 |  |
| Time | 0.025 |  |
| Residual | 0.456 |  |
| **Behavioral well-being** | | |
| **Fixed effects** | **ꞵ coefficients (SE)** | **P-value** |
| Intercept | 3.387 (0.1386) | **<0.0001** |
| Age | -0.04 (0.002) | **<0.0001** |
| Time | 0.15 (0.0135) | **<0.0001** |
| Sex | -0.313 (0.0362) | **<0.0001** |
| Age*time | -0.002 (0.0002) | **<0.0001** |
| Sex*time | 0.008 (0.0033) | **<0.0001** |
| **Random effects** |  |  |
| Intercept | 0.563 |  |
| Time | 0.034 |  |
| Residual | 0.357 | |
| **Psychological well-being** | | |
| **Fixed effects** | **ꞵ coefficients (SE)** | **P-value** |
| Intercept | 1.987 (0.1667) | **<0.0001** |
| Age | -0.025 (0.0024) | **<0.0001** |
| Time | 0.139 (0.0161) | **<0.0001** |
| Sex | -0.072 (0.0435) | **<0.0001** |
| Age*time | -0.003 (0.0002) | **<0.0001** |
| Sex*time | 0.006 (0.0038) | **<0.0001** |
| **Random effects** |  |  |
| Intercept | 0.661 |  |
| Time | 0.022 |  |
| Residual | 0.454 | |
| **Social well-being** |  |  |
| **Fixed effects** | **ꞵ coefficients (SE)** | **P-value** |
| Intercept | 0.758 (0.0986) | **<0.0001** |
| Age | -0.007 (0.0014) | **<0.0001** |
| Time | 0.078 (0.0083) | **<0.0001** |
| Sex | -0.05 (0.0257) | **<0.0001** |
| Age*time | -0.001 (0.0001) | **<0.0001** |
| Sex*time | -0.002 (0.002) | **<0.0001** |
| **Random effects** |  |  |
| Intercept | 0.413 |  |
| Time | 0.019 |  |
| Residual | 0.226 | |

*Note:* SE = standard error.
